# The Feasibility of an App-Based Worksite Health Promotion Program to Improve Mental Well-Being and Work-Related Vitality in University Hospital Workers: Process and Preliminary Effect Evaluation Study

**DOI:** 10.2196/85135

**Published:** 2026-06-17

**Authors:** Sanne H M Kremers, Joline W J Beulens, Cédric N H Middel, Petra J M Elders, Allard J van der Beek, Femke Rutters

**Affiliations:** 1 Health Behaviors and Chronic Diseases, Amsterdam Public Health Research Institute, Amsterdam, The Netherlands; 2 Epidemiology and Data Science, Amsterdam UMC, De boelelaan 1089a, Amsterdam, 1081 HV, The Netherlands, 31 204446759; 3 Julius Centre for Health Sciences and Primary Care, University Medical Centre Utrecht, Utrecht, The Netherlands; 4 Athena Institute, Faculty of Science, Vrije Universiteit Amsterdam, Amsterdam, The Netherlands; 5 General Practice, Amsterdam UMC, Amsterdam, The Netherlands; 6 Public and Occupational Health, Amsterdam UMC, Amsterdam, The Netherlands

**Keywords:** workplace health promotion program, feasibility study, lifestyle, mental well-being, vitality, hospital personnel

## Abstract

**Background:**

University hospital employees face role-specific stressors that can impair mental well-being and work-related vitality. While worksite health promotion programs show potential for improving mental well-being by targeting lifestyle behaviors, most target single professions or hospital subunits, and evidence for mental well-being and work-related vitality remains mixed. Mobile apps offer unique advantages for delivering such worksite health promotion programs hospital-wide. However, accessible interventions tailored to a diverse workforce are lacking.

**Objective:**

This study aimed to investigate the feasibility of an app-based worksite health promotion program (the Recharge360 program [The Recharge Company]) targeting multiple lifestyle behaviors, including a team-based competition element, for improving mental well-being and work-related vitality of hospital employees over a 5-month follow-up period by evaluating two objectives: (1) the implementation process of the program, and (2) the preliminary effects of the program on mental well-being and work-related vitality.

**Methods:**

We included 532 employees (mean age 43, SD 12 y; n=482, 91% women; n=480, 90% highly educated) from a university hospital in Amsterdam, the Netherlands. The study had a single-arm, longitudinal pretest-posttest design lasting 5 months, during which employees participated in the 5-day Recharge360 program (Recharge week) 3 times—in weeks 1, 9, and 17. At baseline (T0) and after each Recharge week (T1-T3), we assessed mental well-being, work ability, need for recovery, and task performance. The process was evaluated by assessing recruitment, attrition, and survey completion rates, and the degree of participation. Preliminary effects were evaluated by linear mixed model regression analyses to assess changes in mental well-being and work-related vitality between baseline and follow-up.

**Results:**

Recruitment appeared feasible, but attrition rates were high (up to 70% in the final Recharge week), and the degree of participation decreased over time. We showed statistically significant, albeit small, increases in well-being at T3 (unstandardized β coefficient=2.08, 95% CI 0.33-3.84), with progressively larger improvements in the analyses among those who started at least 1, 2, and all 3 Recharge weeks (unstandardized β coefficient=3.27, 95% CI 1.09-5.45). Results for work-related vitality were mixed. The need for recovery remained unchanged, task performance increased slightly at T3 (unstandardized β coefficient=0.16, 95% CI 0.07-0.24). Work ability showed a small, but statistically significant, decline across follow-up (unstandardized β coefficient=−0.46, 95% CI −0.64 to −0.29).

**Conclusions:**

This app-based worksite health promotion program might be feasible to implement in a university hospital setting and shows potential to slightly improve mental well-being, but primarily for a selective group of highly educated, health-conscious women. While these findings support further investigation in a randomized controlled trial in similar university hospital settings, they also highlight the need for more participatory study designs to improve the tailoring of program components and engagement of underrepresented groups, as well as for a supportive culture and population-based approaches at the organizational level.

## Introduction

In recent years, the importance of mental well-being and vitality has been increasingly recognized in occupational settings, as it plays a crucial role in both individual and organizational health. Mental well-being fits in a broader context of positive mental health, while vitality is a concept closely related to (mental) well-being and is widely recognized as a positive, energetic state with both mental and physical components [1-3]. In the occupational setting, a mental component of vitality is work-related vitality, which is characterized by high levels of energy and mental resilience while working, the willingness to invest effort in one’s work, and persistence also in the face of difficulties [2,4,5]. Maintaining mental well-being is associated with a lower risk of mental health conditions (eg, depression), chronic diseases (eg, diabetes and cardiovascular diseases), and a higher quality of life [6-8]. Therefore, the consequences of impaired mental well-being are also serious for organizations and society as a whole, as mental health conditions are among the leading causes of sickness absenteeism and work disability in Western European countries and among the leading causes of disease burden worldwide [9,10].

Around 60% of the global population of working age is in paid work, and working adults spend a significant part of their time working [11]. Therefore, the worksite is a convenient setting for health promotion, as it allows for structural and repeated access to large amounts of the adult population, the usage of present social networks, and integration of health promotion via existing organizational infrastructures [12,13]. Worksite health promotion programs targeting healthy lifestyle behaviors, such as abstinence from smoking, sufficient physical activity, healthy dietary habits, relaxation (mental balance), sufficient sleep, and limited alcohol intake, have been developed over the last few decades [14-18]. Several reviews have consistently shown that such programs can potentially improve lifestyle behaviors, mental and physical well-being, work-related vitality, and contribute to the prevention and management of chronic diseases [16,19-25]. Moreover, promoting employees’ well-being and vitality with such programs is also valuable for employers, as it can help to reduce both sickness absenteeism and presenteeism, and improve productivity and work ability [12,17].

Health care workers face role-specific stressors (eg, high workloads, shift work, demands from patient care, and secondary trauma) that can undermine their physical and mental well-being and vitality, contributing to elevated rates of mental disorders, burnout, and corresponding sickness absenteeism, compared with workers holding other jobs [26-28]. In (university) hospital settings, office workers (eg, academic researchers, university, and support staff) may also experience specific stressors, such as competing teaching and scientific research demands, publication pressure, job insecurity, and competition [29].

Worksite health promotion programs for health care workers have shown potential to improve lifestyle behaviors, but results for (mental) well-being and work-related vitality are mixed [30], and most interventions target either single professions (eg, nurses) or subunits within the hospital, instead of the whole hospital. Traditional in-person worksite health promotion programs may not accommodate the diverse needs, routines, and work environments of various hospital workers, reducing the feasibility of such programs [31]. Delivering worksite health promotion through a mobile app can be feasible for hospital settings due to the ability to reach large target groups, location and time independence, 24-hour availability, adaptability to user needs, self-monitoring functions, and cost-effectiveness [31,32]. Also, including social features, such as team-based competition, may enhance users’ engagement with worksite health promotion apps [33,34].

To our knowledge, no studies have specifically examined easily accessible app-based programs targeting the broader university hospital workforce and tailored to its diverse professional groups. Therefore, the overall aim of this study is to investigate the feasibility of a mobile app–based worksite health promotion program (the Recharge360 program [The Recharge Company]) targeting multiple lifestyle behaviors, including a team-based competition element, for improving mental well-being and work-related vitality of hospital employees over a 5-month follow-up period. This was done by performing a pilot and feasibility study aimed at evaluating two objectives: (1) the implementation process of the Recharge360 program, and (2) the preliminary effects of the Recharge360 program on mental well-being and work-related vitality.

## Methods

### Study Design

This study was performed in employees of the Amsterdam University Medical Center (UMC), which is a Dutch university hospital employing around 19,500 employees divided over approximately 80 departments and 2 locations across Amsterdam, the Netherlands. Our study consisted of a pilot study in 1 health care department and 1 office department (n=34) and a larger-scale main study in which all Amsterdam UMC employees could participate (n=532). In the pilot study, we investigated to what extent the Recharge360 program was implemented according to protocol and what facilitators and barriers could contribute to improving future implementation based on a mixed methods process evaluation. These results were then used to improve the implementation of the main study (pilot study process evaluation methods and results are provided in Multimedia Appendix 1). For objective 1, the implementation process was evaluated based on both an evaluation of the pilot study process and an assessment of feasibility parameters in the main study, including recruitment rates, attrition rates, survey completion rates, and degree of participation. For objective 2, preliminary effects were evaluated based on pretest-posttest evaluations in the main study.

This study had a single-arm, longitudinal pretest-posttest design with a duration of approximately 5 months (20 wk), during which all participants were asked to participate in the 5-day Recharge360 program 3 times—in week 1 (first Recharge week), week 9 (second Recharge week), and week 17 (third Recharge week). Before the first Recharge week, participants completed a baseline questionnaire (T0) assessing sociodemographic characteristics, lifestyle, mental well-being, and work-related vitality. Follow-up assessments of mental well-being and work-related vitality were conducted 2 weeks after the first Recharge week (T1, in week 3) to assess short-term outcomes, and 3 weeks after the second (T2, in week 12) and third Recharge weeks (T3, in week 20) to assess longer-term outcomes.

### Participants and Recruitment

Recruitment strategies included 2 digital news articles on the intranet, flyers at diverse locations, posters at diverse locations, and an option to invite a colleague. Employees who provided their email in response to these recruitment strategies were provided with the study information materials. They then had a 2-week period to decide whether to participate in the study.

Eligibility criteria for participating in this study were all employees of Amsterdam UMC who (1) are able and willing to provide written consent to participate, (2) are older than 18 years, (3) are proficient in Dutch or English language, and (4) have access to a smartphone.

### Ethical Considerations

The protocol of this study was submitted to and reviewed by the Medical Ethical Review Committee of the Amsterdam UMC, location Vrije Universiteit (application 2022.0362). This committee has approved the study and established that this research does not fall under the scope of the Medical Research Involving Human Subjects Act and thus was exempted from further evaluation as such. Before participating in this study, all participants provided informed consent. Participants were thoroughly informed about the study and of their study rights, including the right to withdraw from the study. All study data were deidentified before analyses and managed using unique participant identifiers. All data were stored securely on electronic servers. Participants did not receive compensation for participation.

### The Recharge360 Program

The Recharge360 program spans 5 working days (1 work week) and is delivered through an easy-to-use mobile app (Recharge360 app), which can be downloaded on participants’ private phones. It is designed to promote healthy routines by stimulating small behavioral changes and targets multiple lifestyle components, including nutrition, alcohol use, physical activity, relaxation (mental balance), stress, and sleep, and incorporates a team-based competition. The program is based on experiential learning, the process of learning through experience (eg, hands-on and practical experiences combined with theoretical components), and uses existing and widely used health advice [35].

Multimedia Appendix 2 provides a more detailed description of the Recharge360 application and its components. The program provides daily advice for healthy morning, afternoon, and evening routines with accompanying exercises and challenges, that participants can follow voluntarily and without sequential completion requirements. Examples include drinking more fluids, eating more fruit, adopting focus blocks, and mindfulness exercises. Points were assigned to all program components and were collected for individuals, and if chosen to join a team, also for team-based groups as part of the competition among these groups. In this way, each individual’s use of the program benefited the whole team. Before the start of the first Recharge week, there was a kick-off event consisting of a motivational information session.

The Recharge360 program has been developed by The Recharge Company and originally targets office workers. In collaboration with The Recharge Company, a tailored Amsterdam UMC version was developed to accommodate the different roles and schedules of workers within a university hospital. Therefore, two program types were available: (1) for health care workers (eg, outpatient clinics and nursing departments), (2) for office workers (eg, research, education, and support departments). All employees could choose the program that best fitted their job type. The versions were identical in structure but included role-specific exercises and challenges; for example, focus blocks for office workers and mindfulness exercises for health care workers to let go of thoughts about negative or stressful situations regarding patient contact. For the health care workers, the program also provided an informational document on night shifts, based on the “Fit for the night” program used in Dutch UMCs, covering balanced nutrition, hydration, sleep hygiene, short recovery periods, and physical activity. Participants could also chat with coaches to discuss questions or concerns.

### Outcome Measures

#### Feasibility Parameters

To assess the feasibility of recruiting and retaining participants, recruitment rates (enrolled participants/interested employees) and attrition rates (participants lost to follow-up/enrolled participants) were examined. Survey completion rates (participants who completed the follow-up assessment/enrolled participants) were evaluated to examine whether participants were willing to engage in follow-up assessments. Willingness to comply with the program was assessed through the degree of participation in the Recharge360 program. Employees were marked as participating in the program if they started a Recharge week based on data from the Recharge360 app. The degree of participation was then assessed by the number of Recharge weeks participated in (maximum of 3) and the score achieved in the Recharge360 app by completing program components in each separate Recharge week, and for all 3 Recharge weeks in total. The maximum score that could be obtained for 1 Recharge week was 164 (health care version) or 161 (office version).

#### Mental Well-Being

At baseline and all 3 follow-up measurements, mental well-being was measured using the World Health Organization-Five Well-Being Index (WHO-5) [36]. This self-report questionnaire consists of 5 items on a 6-point Likert scale (5=all of the time, 4=most of the time, 3=more than half of the time, 2=less than half of the time, 1=some of the time, and 0=at no time) and asks back over the past 2 weeks, for example, “I have felt cheerful and in good spirits” and “I have felt calm and relaxed.” All items are summed to obtain a raw total score between 0 and 25, which is multiplied by 4 to obtain a percentage score from 0 (worst possible well-being) to 100 (best possible well-being). Generally, a score of ≤50 indicates poor mental well-being and further assessment for the possible presence of a mental health condition, such as depressive disorder [37]. The scale demonstrates sufficient construct and content validity regardless of the presence or absence of illness and in a variety of different settings [37,38].

#### Work-Related Vitality

At baseline and all 3 follow-up measurements, work-related vitality was estimated by measuring work ability, need for recovery, and task performance. Work ability was estimated by the first question of the Work Ability Index (WAI) questionnaire [39], which assesses a worker’s current work ability relative to a worker’s lifetime best work ability on a 10-point scale ranging from 0 (completely unable to work) to 10 (work ability at its best). The first item of the WAI correlates moderately to strongly with the WAI, with Spearman rank correlations ranging from 0.72 to 0.76, and it has been proposed feasible to replace the WAI in occupational health research (eg, epidemiological studies) and employee surveys [40]. Need for recovery was assessed using a subscale of the Questionnaire on the Experience and Evaluation of Work [41]. The scale consists of 11 dichotomous items (yes or no) representing the short-term effects of a working day with a score range of 0‐11. This scale is then transformed to a score range of 0‐100, with a higher score indicating a higher recovery need after work. For example, “I find it hard to relax at the end of a working day.” The need for recovery subscale has shown good reliability (Cronbach α=0.86‐0.88), construct validity, and sensitivity to change in the Netherlands [41-43]. Task performance was assessed using the task performance subscale of the Individual Work Performance Questionnaire (IWPQ) [44,45]. The scale consists of 5 items on a 5-point rating scale (“seldom” to “always”) and questions back over 3 months. The IWPQ task performance subscale has good reliability (Cronbach α=0.78), and the IWPQ has good face and structural validity [44-46].

### Other Variables: Sociodemographic, Lifestyle, and Other Characteristics

At baseline, sociodemographic and other characteristics were assessed with a self-report questionnaire, including age (years), sex (man, woman, and other), education level (low, middle, and high), children at home (yes or no), working hours (8‐16, 17‐24, 25‐36, and >36 hours), sickness absenteeism in the past 5 months (0, <9, or ≥10 sick days), experienced stress (high or low), Patient-Reported Outcomes Measurement Information System global mental health and physical health (*t* test scores), self-rated health (poor, fair, good, very good, and excellent), and shiftwork (rotating shifts, night shifts, or neither) [47,48]. Lifestyle factors were assessed with a self-report questionnaire, including BMI (in kg/m^2^) calculated from body weight (kg) and length (m), fruit intake (<2 or ≥2 pieces per day), vegetable intake (<200 or ≥200 grams per day), alcohol intake (≤7 or >7 glasses per week), smoking habits (current, former, or never), sleep duration between 7 and 9 hours (yes or no), sleep quality (very good, fairly good, rather poor, or very poor) and physical activity in metabolic equivalent of task (MET) minutes/week (refer to Multimedia Appendix 3 for a more detailed description of measurement methods) [49-51].

### Sample Size and Power Calculation

A sample size of 588 participants was required to achieve 80% power to demonstrate an absolute difference of 2 points in mental well-being (scale 0‐100) between 2 measurements, with a 2-sided significance level of 5%. This sample size calculation assumed an SD of 18 points in mental well-being; both the minimum expected difference (2 points) and the SD were estimated from available literature [52-54]. The calculation was based on 4 longitudinal measurements with a correlation coefficient of 0.6 (ρ) and an intracluster correlation coefficient of 0.05, taking into account possible correlation between participants from the same department. A dropout rate of 20% during follow-up was considered.

### Statistical Analysis

Statistical analyses were performed using R-studio (version 4.3.2; Posit). A 2-sided *P* value ≤.05 was considered statistically significant. Baseline descriptive characteristics are presented for the total population and for health care and office workers separately. Categorical variables are presented as frequencies (%) and continuous variables as mean (SD) if normally distributed or as median (IQR) if nonnormally distributed after visual assessment of QQ-plots.

We used descriptives to investigate recruitment rates, attrition rates, survey completion rates, and the degree of participation as parameters for program feasibility.

To evaluate preliminary effects, we used linear mixed model regression analyses to assess the change in continuous outcomes (WHO-5 and work-related vitality) between baseline (T0) and at T1 (in week 3), T2 (in week 12), and T3 (in week 20). The time points were included as fixed effects, whereas participant ID was included as a random effect to account for potential within-participant correlations over follow-up. We tested whether the change in outcome over time differed between participants who followed the office program versus the health care program by adding interaction terms to the model. If the interaction term was statistically significant (*P*<.10), analyses were stratified accordingly.

We performed an intention-to-treat analysis, including all participants who started the program, regardless of their withdrawal from the program. Additionally, we performed a per-protocol analysis, including participants who (1) started 1 Recharge week, (2) started 2 Recharge weeks, and (3) started all 3 Recharge weeks. Missing data in outcome measurements were handled by the linear mixed model, which handles missing outcome data automatically by an estimation technique based on a full information maximum likelihood model, and under the assumption of a missing at random mechanism [55]. We compared the characteristics of participants who dropped out by starting 0 Recharge weeks, versus participants who dropped out but still started 1 or 2 Recharge weeks, versus participants who completed the program by starting all 3 Recharge weeks.

## Results

### Population Characteristics

**Figure 1.**
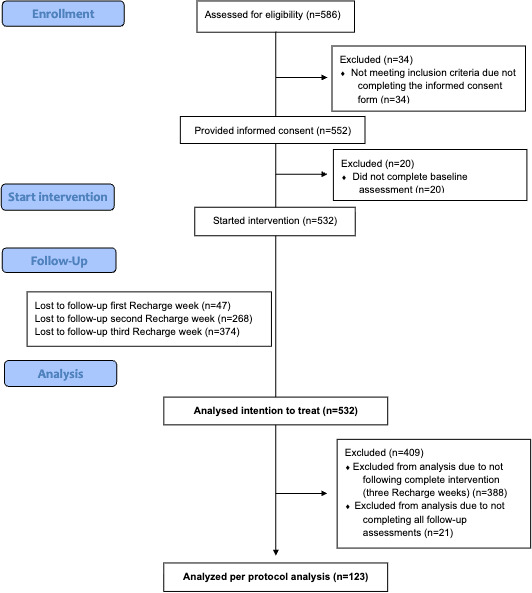
Flowchart of study participants. Reasons for lost to follow-up are provided in Multimedia Appendix 4.

In total, 586 participants were assessed for eligibility by signing up for the study, of whom 34 were excluded due to not providing informed consent and 20 due to not completing the baseline questionnaire (these 20 participants also did not participate in any of the Recharge weeks). This resulted in 532 participants starting the Recharge360 program. The analytic sample was 532 for the intention-to-treat analysis and 123 for the per-protocol analysis (Figure 1).

The total study population consisted of 532 employees of the Amsterdam UMC with a mean age of 43 (SD 12) years, and 91% (482) were women (Table 1). The mean score for mental well-being was 60 (SD 16). Regarding work-related vitality, the mean score was 8 (SD 1.4) for work ability, 41.8 (SD 29.6) for need for recovery, and 2.5 (SD 0.7) for task performance. Comparing office workers (N=360) with health care workers (N=129), health care workers were more often women (125, 97% vs 319, 89%), had a higher need for recovery (46.7 vs 38.7), more often adhered to the Dutch Dietary Guidelines 2015 for fruit intake (53, 41% vs 120, 33%) and vegetable intake (49, 38% vs 92, 26%), less often drank >1 alcoholic beverage per day on average (12, 9% vs 46, 13%), and had higher physical activity (2775 vs 2034 MET minutes/week). Health care workers also had an optimal sleep duration of 7-9 hours per day less often (87, 67% vs 266, 74%) and more often performed shift work (46, 36% vs 6, 2%).

**Table 1.** Baseline characteristics of 532 participants from the Amsterdam UMC challenge (visual assessment of QQ-plots).

Characteristics	Total population (n=532)	Office program (n=360)^a^	Health care program (n=129)^a^
Demographics			
Age (y), mean (SD)	43 (12)	43 (12)	41 (11)
Women, n (%)	482 (90.6)	319 (88.6)	125 (96.9)
High education level, n (%)	480 (90.2)	327 (90.8)	118 (91.5)
Other characteristics			
Children at home (yes), n (%)	247 (46.4)	158 (43.9)	64 (49.6)
Working hours, n (%)			
≤24 h	56 (10.6)	31 (8.6)	18 (20)
25-36 h	362 (68)	250 (69.4)	88 (68.2)
>36 h	114 (21.4)	79 (21)	23 (17.8)
High stress, n (%)	262 (49.2)	163 (45.3)	69 (53.5)
Global physical health (t-score), mean (SD)	50.0 (4.56)	49.7 (4.54)	51.1 (4.62)
Global mental health (t-score), mean (SD)	46.9 (3.70)	47.1 (3.70)	46.6 (3.70)
Self-rated health, n (%)			
Poor or fair	103 (19.4)	68 (18.9)	22 (17.1)
Good	226 (42.5)	162 (45)	48 (37.2)
Very good or excellent	203 (38.1)	130 (36.1)	59 (45.7)
Well-being			
WHO-5^b^, score (range 1-100), mean (SD)	60 (16)	60 (15)	61 (17)
Work-related vitality			
Work ability, score (range 1-10), mean (SD)	8 (1.4)	8 (1.3)	8 (1.2)
Sickness absenteeism last 5 months (≥9 d), n (%)	24 (4.5)	14 (3.9)	8 (6.2)
Need for recovery (range 1-100), score, mean (SD)	41.8 (29.6)	38.7 (28.3)	46.7 (30.8)
Task performance score (range 1-4), mean (SD)	2.5 (0.7)	2.5 (0.7)	2.5 (0.8)
Lifestyle			
Smoking behavior, n (%)			
Current	26 (4.9)	18 (5)	5 (3.9)
Former	120 (22.6)	93 (25.8)	21 (16.3)
Never	386 (72.6)	249 (69.2)	103 (79.8)
Electronic smoking behavior, n (%)			
Current	7 (1.3)	4 (1.1)	3 (2.3)
Former	17 (3.2)	12 (3.3)	3 (2.3)
Never	508 (95.5)	344 (95.6)	123 (95.3)
BMI (kg/m^2^)^c^, mean (SD)	24.8 (4.7)	25.1 (4.9)	24.1 (4.1)
Vegetable intake (≥200 g/d), n (%)	153 (28.8)	92 (25.6)	49 (38)
Fruit intake (≥2 pieces/day), n (%)	190 (35.7)	120 (33.3)	53 (41.1)
Alcohol intake (>7 glasses/wk), n (%)	68 (12.8)	46 (12.8)	12 (9.3)
Sleep duration (7-9 h/d), n (%)	381 (71.6)	266 (73.9)	87 (67.4)
Overall sleep quality (fairly good or very good), n (%)	403 (75.8)	278 (77.3)	99 (76.7)
Shifts during the past month^d^, n (%)			
Rotating and/or night shifts	61 (11.5)	6 (1.7)	46 (35.9)
Neither	470 (88.5)	354 (98.3)	82 (64.1)
Physical activity (MET^e^; min/wk)^f^, median (IQR)	2118 (1158-3576)	2034 (1103-3499)	2775 (1372-4584)

a Some participants (n=43) only completed the questionnaire and did not follow the program (neither health care program nor office program). Therefore, those participants are not included in these 2 columns.

b WHO-5: World Health Organization-Five Well-Being Index.

c Analytic sample for BMI was n=528.

d Analytic sample for shifts during the past month was n=531.

e MET: metabolic equivalent of task.

f Analytic sample for metabolic equivalent of task minutes/week was n=369.

People who started at least 1 Recharge week (N=345) were fairly similar to those who completed all 3 Recharge weeks (N=114), except that completers were more often office workers (119, 83% vs 241, 70%) (Multimedia Appendix 5). Compared with people who did not start any Recharge weeks (N=43), people who completed all 3 Recharge weeks were more often highly educated (134, 93% vs 35, 81%), less often had children at home (70, 49% vs 25, 58%), less often worked ≤24 hours (13, 9% vs 7, 16%) or >36 hours (33, 23% vs 12, 28%) per week, less often had high stress (60 42% vs 30, 70%), had a slightly higher WHO-5 (60 vs 56), had a lower need for recovery (38.4 vs 53.1), less often drank more than 7 glasses of alcohol per week (16, 11% vs 10, 23%), more often had a fairly or very good sleep quality (108, 75% vs 26, 61%), more often did no shift work (132, 92% vs 34, 79%), and had lower physical activity (2132 vs 2360 MET minutes/week).

### Feasibility

#### Recruitment Rate

Of around 19,500 Amsterdam UMC employees, 892 employees provided their email for more information, in response to the recruitment strategies, of whom 586 signed up to participate, and 532 completed the informed consent form and baseline assessment (recruitment rate=60%).

#### Attrition Rates Recharge360 Program and Survey Completion Rates

Regarding attrition rates, of the 532 participants, 50 did not start the first Recharge week (attrition rate=9%), 268 participants did not start the second Recharge week (attrition rate=50%), and 374 participants did not start the third Recharge week (attrition rate=70%) (Figure 1).

Regarding survey completion, of the 532 participants, 405 (76%) completed the first follow-up assessment (T1), 261 (49%) completed the second follow-up assessment (T2), and 197 (37%) completed the third follow-up assessment (T3). A total of 123 (23%) participants started all Recharge weeks and completed all questionnaires. Only 29 participants formally dropped out of the study by emailing the investigators and providing a reason for dropout (Multimedia Appendix 4).

#### Degree of Participation in the Recharge360 Program (Compliance)

The degree of participation declined over time, as shown by the decreasing number of participants starting each Recharge week. This is also reflected in the attrition rates. A total of 144 (27%) participants complied with the complete program by starting all 3 Recharge weeks. Overall, in those starting a Recharge week, the degree of participation (reflected by the mean score per week) was quite consistent for each Recharge week (score ≥60), although highest in the first Recharge week (median 71, IQR 37-92; Table 2). More office program participants started all 3 Recharge weeks (119/360, 33%), compared with health care participants (25/129, 19%). Although office program participants started more Recharge weeks, their degree of participation in each Recharge week was lower compared with health care workers (Table 2). Among office workers, the degree of participation was highest during the first Recharge week, whereas for health care workers, it was highest in the last Recharge week.

**Table 2.** Degree of participation in each Recharge week.

Department	Recharge week 1		Recharge week 2		Recharge week 3		All weeks^a^	
	n	Score, median (IQR)	n	Score, median (IQR)	n	Score, median (IQR)	n	Score, median (IQR)
Health care^b^	127	73 (30-95)	58	71 (31-106)	29	87 (66-104)	129	83 (29-158)
Office^c^	355	70 (42-91)	206	59 (25-88)	129	59 (27-81)	360	92 (50-179)
Both	482	71 (37-92)	264	60 (28-91)	158	63 (33-86)	489	90 (44-177)

a The sum of the median score over all weeks in people that started at least one recharge week.

b The maximum score that could be obtained for 1 Recharge week was 164.

c The maximum score that could be obtained for 1 Recharge week was 161.

#### Usage of Other Application Functions

A total of 64 teams started in the first, 48 in the second, and 45 in the third Recharge week, with each team representing different departments or subteams within departments. In the first, second, and third Recharge weeks, 135, 19, and 5 participants viewed the nutrition guide; 57, 6, and 3 viewed the parents’ guide; and 35, 6, and 3 viewed the night workers’ guide, respectively (mostly 1 or 2 times). Moreover, 0 participants used the function to contact a coach via the app to discuss questions or concerns.

### Mental Well-Being

In the intention-to-treat analyses, the WHO-5 was significantly lower by 1.35 (95% CI −2.68 to −0.02) points at T1, but significantly higher by 2.08 (95% CI 0.33-3.84) points at T3 (Table 3).

**Table 3.** Change in WHO-5^a^ T1 (after 1 Recharge week), T2 (after 2 Recharge weeks), and at T3 (after 3 Recharge weeks) for intention-to-treat and per-protocol analyses. Missing data in outcome measurements were handled by the linear mixed model.

WHO-5 (range 0‐100)	n	Baseline T0	T1, unstandardized β coefficient (95% CI)	T2, unstandardized β coefficient (95% CI)	T3, unstandardized β coefficient (95% CI)
Intention-to-treat^b^	532	Reference	−1.35 (−2.68 to −0.02)^c^	0.76 (−0.81 to 2.33)	2.08 (0.33 to 3.84)^c^
Per-protocol ≥1 Rweeks^d,e^	489	Reference	−1.32 (−2.63 to 0.002)	0.53 (−1.03 to 2.08)	2.06 (0.32 to 3.80)^c^
Per-protocol ≥2 Rweeks^f^	271	Reference	−0.37 (−2.05 to 1.31)	1.29 (−0.50 to 3.08)	2.55 (0.65 to 4.67)^c^
Per-protocol all Rweeks^g^	144	Reference	0.87 (−1.24 to 2.97)	2.61 (0.48 to 4.74)^c^	3.27 (1.09 to 5.45)^c^

a WHO-5: World Health Organization-Five Well-Being Index.

b Missing data in outcome (N=532): T1, n=127 (24%); T2, n=271 (51%); T3, n=335 (63%).

c Statistically significant findings.

d Rweek: Recharge week.

e Missing data in outcome (N=489): T1, n= 91 (19%); T2, n=231 (47%); T3, n=298 (61%).

f Missing data in outcome (N=271): T1, n=19 (7%); T2, n=59 (22%); T3, n=96 (35%).

g Missing data in outcome (N=144): T1, n=2 (1%); at T2, n=7 (5%); T3, n=16 (11%).

The WHO-5 significantly increased at T3 in all per-protocol analyses, with 2.06 (95% CI −2.68 to −0.02) points, 2.55 (95% CI 0.65-4.67) points, and 3.27 (95% CI 1.09-5.45) points in people who started at least 1, at least 2, or all 3 Recharge weeks, respectively (Table 3). In the per-protocol analysis of people who started all 3 Recharge weeks, but not in those of people who started at least 1 or 2 Recharge weeks, the WHO-5 was also significantly increased with 2.61 (95% CI 0.48-4.74) points at T2. The change over time did not differ by type of program (office vs health care; all *P*-for-interaction >.10).

### Work-Related Vitality

In the intention-to-treat analyses, work ability was significantly lower at T1 (unstandardized β coefficient=−0.52, 95% CI −0.65 to −0.39), T2 (unstandardized β coefficient=−0.50, 95% CI −0.65 to −0.35), and T3 (unstandardized β coefficient=−0.46, 95% CI −0.64 to −0.29), but effect sizes were small (Table 4). No significant changes were found regarding the need for recovery over all follow-up moments. Task performance was significantly higher at T3 (unstandardized β coefficient=0.10, 95% CI 0.04-0.17), but again the effect size was small.

**Table 4.** Change in work-related vitality at T1 (after 1 Recharge week), at T2 (after 2 Recharge weeks), and at T3 (after 3 Recharge weeks) for intention-to-treat analysis and per-protocol analyses. Missing data in outcome measurements were handled by the linear mixed model.

Work-related vitality	n	Baseline T0	T1, unstandardized β coefficient (95% CI)	T2, unstandardized β coefficient (95% CI)	T3, unstandardized β coefficient (95% CI)
Work ability (range 1‐10)					
Intention-to-treat^a^	532	Reference	−0.52 (−0.65 to −0.39)^b^	−0.50 (−0.65 to −0.35)^b^	−0.46 (−0.64 to −0.29)^b^
Per-protocol ≥1 Rweeks^c,d^	489	Reference	−0.52 (−0.65 to −0.39)^b^	−0.51 (−0.67 to −0.36)^b^	−0.49 (−0.66 to −0.32)^b^
Per-protocol ≥2 Rweeks^e^	271	Reference	−0.51 (−0.68 to −0.35)^b^	−0.47 (−0.64 to −0.29)^b^	−0.48 (−0.67 to −0.29)^b^
Per-protocol all Rweeks^f^	144	Reference	−0.51 (−0.70 to −0.31)^b^	−0.31 (−0.51 to −0.11)^b^	−0.44 (−0.64 to −0.23)^b^
Need for recovery (range 1‐100)					
Intention-to-treat^a^	532	Reference	−0.22 (−1.99 to 1.55)	1.18 (−0.94 to 3.29)	−1.58 (−3.93 to 0.76)
Per-protocol ≥1 Rweeks^d^	489	Reference	−0.12 (−1.89 to 1.66)	1.41 (−0.70 to 3.52)	−1.20 (−3.55 to 1.16)
Per-protocol ≥2 Rweeks^e^	271	Reference	−1.11 (−3.35 to 1.13)	0.89 (−1.51 to 3.29)	−1.51 (−4.06 to 1.04)
Per-protocol all Rweeks^f^	144	Reference	−1.35 (−3.19 to 2.49)	0.20 (−2.67 to 3.09)	−1.53 (−4.48 to 1.41)
Task performance (range 1‐4)					
Intention-to-treat^a^	532	Reference	−0.04 (−0.09 to 0.01)	0.004 (−0.06 to 0.07)	0.10 (0.04 to 0.17)^b^
Per-protocol ≥1 Rweeks^d^	489	Reference	−0.04 (−0.09 to 0.01)	0.003 (−0.06 to 0.07)	0.11 (0.04 to 0.17)^b^
Per-protocol ≥2 Rweeks^e^	271	Reference	−0.05 (−0.11 to 0.02)	−0.01 (−0.08 to 0.06)	0.10 (0.02 to 0.17)^b^
Per-protocol all Rweeks^f^	144	Reference	−0.01 (−0.09 to 0.07)	0.004 (−0.08 to 0.09)	0.16 (0.07 to 0.24)^b^

a Missing data in outcome (N=532): T1, n=132 (25%); T2, n=282 (53%); T3, n=337 (63%).

b Statistically significant findings.

c Rweek: Recharge week.

d Missing data in outcome (N=489): T1, n=92 (19%); T2, n=233 (48%); T3, n=300 (61%).

e Missing data in outcome (N=271): T1, n=19 (7%); T2, n=59 (22%); T3, n=97 (36%).

f Missing data in outcome (N=144): T1, n=2 (1%); at T2, n=7 (5%); T3, n=17 (12%).

In the per-protocol analysis, work ability was significantly lower at all follow-up moments for all analyses, but effect sizes were small (ranging from unstandardized β coefficient=−0.31, 95% CI −0.51 to −0.11 to unstandardized β coefficient=−0.52, 95% CI −0.65 to −0.39; Table 4). No statistically significant changes were found regarding the need for recovery over time in any of the analyses. Task performance was significantly higher at T3 for all analyses, but again, the effect size was small. The highest increment was in the analyses of people who started all 3 Recharge weeks, with an increase of 0.16 (95% CI 0.07-0.24) points. The change over time did not differ by type of program (office vs health care; all *P*-for-interaction >.10).

## Discussion

### Principal Findings

This study investigated the feasibility of a mobile app–based worksite health promotion program with a team-based competition element (the Recharge360 program) for improving mental well-being and work-related vitality in university hospital employees over a 5-month follow-up period based on a process and preliminary effect evaluation. The process evaluation showed that recruitment was feasible, but attrition rates were high, and the degree of participation decreased over time. Overall, the preliminary effect evaluation suggests feasibility for improving mental well-being, with significantly higher scores at T3 (after 3 Recharge weeks). However, we found mixed results for work-related vitality, ranging from a significant, small decrease in work ability to an increase in task performance.

Recruitment was feasible in terms of numbers, and the team component was successfully integrated. Despite this, the study sample was selective, consisting mostly of nonsmoking, highly educated women with, on average, a normal BMI, and quite good baseline scores for mental well-being, work ability, and need for recovery. This may partly be explained by self-selection, also known as the healthy volunteer effect, with participants in research and health-related programs being typically healthier, more highly educated, and more health-conscious than nonparticipants [56-59]. The additional demands of the research component, such as signing an informed consent and the time necessary to fill out questionnaires, may have further affected recruitment rates and amplified this effect compared with participation in low-threshold workplace health promotion programs.

Another finding is that attrition rates were high and increased over time (70% in the final Recharge week), suggesting limited feasibility for retaining program participants. In the literature, attrition rates of digital worksite health promotion programs vary widely, and comparison is difficult due to a wide variation in intervention duration, definitions of attrition, delivery modes, and population characteristics. Still, the overall picture is that participation rates are often below 50% and different mobile and online worksite health programs with similar goals have shown varying attrition rates between 0% and 75% [60-63]. Only 5% (29/532) of participants formally dropped out with a provided reason, most often mentioning inconvenient timing (eg, due to a holiday or too little time), followed by personal circumstances, and to a lesser extent, issues with the program itself. However, no consistent patterns in underlying reasons were identified due to most participants dropping out quietly, offering limited guidance for improving retention. Problems with the timing of the program suggest a need for flexible scheduling of the Recharge weeks, which the program already allows, but was standardized for research purposes. Moreover, not having enough time to participate needs ongoing attention on an organizational level; for example, support of direct supervisors and managers and a supportive organizational culture are known to be important for participation in workplace health promotion programs [34]. This is in line with our pilot process evaluation, indicating that department culture could be both a contextual facilitator (eg, already existing attention for lifestyle behaviors and supportive collegial environment) or barrier (eg, busy and demanding work schedules) for participation.

The degree of participation in the program via the app declined slightly over time, similarly to other worksite health promotion apps [32]. Participants who did not start the program more often had lower education levels, children at home, lower mental well-being, higher need for recovery, and shift work. Despite additional information materials being available to improve engagement in some of these groups, such as the parents’ guide and advice for night workers guide, they were mostly viewed only once or twice in the first Recharge week, and not by everyone, suggesting that providing additional information, at least in this form, was not effective to improve engagement in these groups. Comparison with other studies is difficult due to variation in measures, intervention types, and follow-up periods. Still, one study of 6 months among health care workers using online mental health modules found that those who dropped out had reduced work-related vitality (work functioning and engagement), while a study investigating a 6-week app-based resilience training found no differences between completers and noncompleters [60,64,65].

Regarding preliminary effects, the program appears feasible to improve mental well-being, with small but relevant increases over follow-up on a population level. The smallest increase was observed in the analysis among those who started at least 1 Recharge week, and progressively larger improvements were found in the analyses among those who completed at least 2 and all 3 Recharge weeks. These results were not modified by differences in baseline characteristics, as those were fairly similar for people who attended 1 or 2 versus 3 Recharge weeks. This suggests that improving the degree of participation is needed to further explore the effect of this program. Overall, these results align with other worksite health promotion programs showing improvements in mental well-being in (randomized) controlled trials [53,65-68], although not always statistically significant [60,69]. However, a more detailed comparison is difficult as the earlier studies used a range of different intervention durations, delivery methods (eg, in-person, web-based, or mobile app), intervention targets (eg, a single lifestyle behavior or mental health rather than multiple lifestyle targets), populations (eg, only nurses), and study designs (eg, nonrandomized and randomized controlled trials).

Results for work-related vitality were mixed. While the need for recovery remained unchanged and task performance increased slightly at T3, work ability showed a small but statistically significant decline across follow-up. Given the high work ability baseline level, a ceiling effect may have limited potential for improvements. These findings are not in line with some randomized controlled trials investigating other worksite health promotion programs that showed no significant effect on work ability [64,67,69,70] and significantly improved need for recovery [53,71]. Again, however, these results are difficult to compare due to the same reasons as mentioned for mental well-being. One of these studies found no effect on the first item of the WAI measuring current relative to lifetime best work ability (as used in this study), but did find effects on other WAI items, including work ability with respect to physical and mental job demands [70]. Although the first WAI item has been proposed to be feasible to replace the complete WAI in occupational health research [40], it might be necessary to measure multiple relevant WAI items to elucidate these conflicting results. One of the trials with an intervention duration of 9 months also proposed that perceived work ability might be somewhat insensitive to shorter-term changes [67].

Strengths of this feasibility study are the elaborate design, including a process evaluation and a preliminary effect evaluation based on both a preparatory pilot phase and a larger-scale main study with multiple follow-up measurements over 5 months. This provides important insights for further improving the feasibility and evaluating the effectiveness of similar worksite health promotion programs. However, some important limitations should be noted. First, the selective study sample limits the generalizability of the findings to the broader population of university hospital employees and might cause bias in interpreting the results, potentially overestimating potential effects, as healthier and more motivated individuals are more likely to participate and benefit. Nevertheless, the results would be comparable with other worksite health promotion studies, which likely face similar selection biases. Second, preliminary analyses were limited by the substantial amount of missing data at follow-up, which increased over time up to 61% at T3. Although the longitudinal linear mixed model regression analyses handle missing data under the missing at random assumption, bias cannot be fully ruled out, and the findings should be interpreted with caution. Third, this study used a nonrandomized single-arm pretest-posttest design without control group, and thus, no causality can be inferred in the preliminary effect evaluation.

### Future Directions

The positive changes in mental well-being support the need for further investigation in a fully powered cluster-randomized controlled trial to confirm these findings and to clarify the mixed results for work-related vitality. However, some improvements should be taken into account. First, improved tailoring of program components to accommodate the diverse needs of participants, particularly those who were more likely not to start the program (eg, people with children and people who do shift work), might be a good strategy to reduce attrition and improve the degree of participation. For example, the program could be improved by tailoring specific program components (eg, exercises and challenges) to these groups instead of offering separate information guides and providing more options to adapt to personal circumstances and schedules. Second, to reach a more diverse audience, recruitment strategies and program components could be adapted to better engage underrepresented groups (eg, men and more practically trained staff), which could be further investigated by including these groups in the pre-execution phase. However, it could also mean that this program is primarily suitable for the current population and is therefore best implemented within that group. A potential strategy to improve both tailoring of program components and engagement of underrepresented groups is adopting a more participatory design, such as using cocreation, which involves nonacademic actors throughout the research process to enhance the relevance, acceptability, and impact of such programs [72].

App-based worksite health promotion programs, such as the Recharge360 program, can be meaningful within a hospital setting as a feasible and accessible initiative offered through human resources or occupational health services, supporting employee well-being and vitality for some groups in a preventive way. This can also contribute positively to the hospital’s image as an employer that wants to improve the mental well-being and vitality of its employees. However, to achieve sustainable improvements in well-being and vitality, support from and intervention at multiple organizational levels are required. This includes population-based approaches that improve the food, physical activity, and psychosocial environments of workplaces [17,23,73-75].

### Conclusion

In conclusion, this app-based worksite health promotion program might be feasible to implement in a university hospital setting and shows potential to slightly improve mental well-being, but primarily for a selective group of highly educated, health-conscious women. While these findings support further investigation in a randomized controlled trial in similar university hospital settings, they also highlight the need for more participatory study designs to improve tailoring of program components and engagement of underrepresented groups, as well as for a supportive culture and population-based approaches at the organizational level.

## Supplementary material

10.2196/85135 Multimedia Appendix 1 Pilot study methods and results.

10.2196/85135 Multimedia Appendix 2 Supplemental methods Recharge360 application.

10.2196/85135 Multimedia Appendix 3 Supplemental methods measurement of sociodemographic, lifestyle, and other characteristics.

10.2196/85135 Multimedia Appendix 4 Reasons for lost to follow-up (n=29).

10.2196/85135 Multimedia Appendix 5 Comparison of participants who completed the whole program vs participants who dropped out.

## Abbreviations

IWPQ: Individual Work Performance Questionnaire
MET: metabolic equivalent of task
UMC: University Medical Center
WAI: Work Ability Index
WHO-5: World Health Organization-Five Well-Being Index
