# Supplementary material for: The Feasibility of an App-Based Worksite Health Promotion Program to Improve Mental Well-Being and Work-Related Vitality in University Hospital Workers: Process and Preliminary Effect Evaluation Study
Source: JMIR Form Res. 2026 Jun 17;10:e85135. doi: 10.2196/85135 (PMC13274912; doi:10.2196/85135)
Supplement: Multimedia Appendix 1 [file formative-v10-e85135-s001.docx]

**Appendix 1: pilot study methods and results**

**Appendix 1.1. Pilot study and its process evaluation methods**

As described in the main paper, before the start of the main study, we performed a pilot study and process evaluation to evaluate to what extent the Recharge360 program was implemented according to protocol and to identify what factors (facilitators and barriers) could contribute to improving implementation. For this purpose, we performed a similar study as the main study, with a duration of 5 months among 35 Amsterdam University medical Centre (UMC) employees of either a hospital department providing patient care (hereafter referred to as healthcare department) or an research department. Employees from these departments mainly work in a healthcare and office setting, respectively. For this analysis we used a mixed methods approach guided by the Medical Research Council (MRC) framework for the process evaluation of complex interventions, consisting of the domains context, implementation and mechanisms of impact [1, 2]. This process evaluation was designed to measure the context and implementation (fidelity, dose, adaptations and reach). Table 1 provides an overview of the operationalization of the MRC framework domains, indicators and data sources for the current process evaluation.

We performed semi-structured interviews with a pre-determined set of open-ended questions regarding the implementation of the Recharge360 program, based on the key domains of the MRC framework. We recruited individuals of both pilot departments who provided written informed consent to participate in an interview and used purposive sampling to achieve a varied sample in terms of degree of participation and work. Interviews were transcribed en verbatim and then summarized to share with participants for member checking to enhance the validity of the interviews. We performed a deductive thematic analyses of the interviews using Max Qualitative Data Analysis (MaxQDA)[3], guided by the predefined domains from the MRC framework. Semi-open coding was used to identify facilitators and barriers related to each key domain of the framework. Three interviews were double coded to reach consensus about the codes and their definition, strengthening internal validity of the analysis. Following this, the coded segments were examined to identify recurring themes in barriers and facilitators within the predefined MRC framework domains.

Other measurements included questionnaires assessing participant characteristics and outcome measures (at baseline, 2, 11 and 19 weeks), data on usage of the Recharge360 application, and project members’ field notes of relevant team meetings, e-mail contacts and observations (Table 2).

Table 1. Operationalization of the MRC framework for the process evaluation of pilot implementation of the Recharge360 program.

| **Domain** | **Pilot-specific operationalization** | **Indicators** |
| --- | --- | --- |
| **Context: contextual factors that affect the implementation of the Recharge360 program** | | |
| Context | An examination of how external factors and environment related to the context act as a barrier or facilitator to the implementation of the Recharge360 program (e.g., work schedules, relocations of departments, hospital location, department culture, and work ethics). How and to what extent the implementation did vary between contexts (e.g., healthcare versus office setting)? | - Characteristics of the participating departments and variation in contexts - Facilitators and barriers to implementation |
| **Implementation: how the delivery of the Recharge360 program is achieved and what is delivered** | | |
| Fidelity | The extent to which the Recharge360 program was delivered as intended and in accordance with protocol. The quality of the program components of the Recharge360 program delivered/ implemented in practice. | - Intervention program delivered according to protocol - Quality of delivered intervention program - Facilitators and barriers to implementation |
| Dose (delivered and received) | The quantity of the program delivered. Actual participation in the Recharge360 program, including the number of Recharge weeks attended, and the degree of participation in the program components. | - Quantity of intervention delivered and received - Participants' degree of participation - Facilitators and barriers to implementation |
| Adaptations | The extent to which adaptations were made to the Recharge360 program during the pilot implementation and why. Adaptations can be made during the pilot study to fit the Recharge360 program in different contexts and changes can also undermine intervention fidelity. | - Adaptations made and why |
| Reach | Whether the intended audience comes into contact with the Recharge360 program and how. Sociodemographic and health-related characteristics of the participants who were recruited in the program. | - Intended audience reached - Characteristics of the participants - Facilitators and barriers to implementation |
| **Mechanisms of impact:**  **how does the Recharge360 program produce a change in outcomes** | | |
| Mechanisms of impact | An examination of the processes through which the Recharge360 program affects outcomes (well-being, work-related vitality, and lifestyle) through understanding how participants respond to and interact with the Recharge360 program and how the intervention supports change in these outcomes (or not). | This domain is not included in this process evaluation since it mainly examines the underlying mechanisms through which the intervention program affects outcomes. This was not part of the goal of this process evaluation of the pilot implementation of the Recharge360 program. |

Table 2. Overview of data sources for each domain.

|  | **Participants** | | | **Administration office & management team** | **Department  head** | **Project team** |
| --- | --- | --- | --- | --- | --- | --- |
|  | Interview | Baseline questionnaires | Recharge360 app | Interview | Interview | Field notes |
| **Context** | X | X |  | X | X | X |
| **Implementation** |  |  |  |  |  |  |
| Fidelity | X |  |  | X |  | X |
| Dose delivered |  |  | X |  |  | X |
| Dose received | X |  | X | X | X |  |
| Adaptations |  |  |  |  |  | X |
| Reach | X | X |  | X | X | X |

## Appendix 1.2. Pilot study and its process evaluation results

Ten semi-structured interviews lasting up to 30 minutes, including three participants with low participation, four participants with high participation, one department head and two participants from the administration office or management team. Based on the analyses of the coded interviews, we observed the facilitators and barriers described in table 3, categorized according to the MRC framework (context, fidelity, dose, adaptations, reach). The paragraphs below (1 to 6) describe the findings for each domain of the MRC framework based on interviews, questionnaires, data from the Recharge360 app and project team members’ field notes (see Table 2 for an overview of measurement methods per domain).

Table 3. Overview facilitators and barriers emerging from the interviews (n=10).^a^

| **Domain** | **Facilitators** | **Barriers** |
| --- | --- | --- |
| **Context** | Department culture | - Financial burden of healthy foods - Department relocation - Department culture - Personal circumstances |
| **Implementation - fidelity** | - App functionality and user experience - Valuable program content - Accessibility of program components - Team aspect and competition - Clear information materials and communication | - Repetitiveness of program components - Practical issues and time investment for cooking recipes - Practical issues and time investment mindfulness exercises - Unrealistic program components and competition - Anonymity - Amount and clearness of information - Technical issues application |
| **Implementation - dose** | - Suitable duration of activities and questionnaires - Good frequency of communication materials - Well-distributed activities | - Spread of Recharge weeks - Lack of (personal) communication in between Recharge weeks - Lengthy activities and questionnaires |
| **Implementation - reach** | Contact with the project team and departmental e-mails | Passive and formal recruitment strategies |
| **Implementation - adaptations** | Not applicable^b^ | Not applicable^b^ |
| **Other factors** | Easy and valuable contact with the project team |  |
| 1. More detailed explanations of these facilitators and barriers for each domain are provided in paragraph 1.2., 2.2., 3.2., 4.2., 6.2. 2. Not applicable. No facilitators and barriers were evaluated for adaptations, since this domain was analysed based on project team field notes. | | |

1. Context

*1.1.* *Characteristics of the participating departments and variation in contexts*

The healthcare department consisted of 165 employees and of the research department of 65 employees, with both departments operating in smaller teams divided over two work locations across Amsterdam. Thirty-one employees of both departments with diverse professions participated in the pilot study (Table 4*).* Before the start of the pilot study, the implementation was already adapted to the different contexts, in this case healthcare vs. office setting, by modifying the wording of communication materials slightly to better fit the context and by running two versions of the program (healthcare and office version).

Table 4. The professions of the participants in the pilot study.

| Professions | Research departement  n=25 | Healthcare departement  n=9 |
| --- | --- | --- |
| Scientific research support staff (e.g. data managers, research assistant) | 4 | 0 |
| Manager or coördinator | 4 | 1 |
| Medical specialist (or in training) | 0 | 3 |
| Scientific researchers (e.g. PhD candidate and post-doctoral researchers) | 15 | 1 |
| Professor | 1 | 0 |
| Administration | 1 | 3 |
| Specialist nurse | 0 | 1 |

*1.2. Contextual facilitators and barriers* *in the pilot implementation*

A key contextual facilitator was department culture. Participants of both departments described participating in the Recharge360 program as a valuable addition as there was already focus on certain topics, such as nutrition and physical activity. Also, the sense of collegiality and involvement within the department helped inclusions. Additionally, the vision that participation could be balanced within regular work responsibilities, indicated an accommodating environment for engagement.

Identified contextual barriers included the financial burden of healthy foods, department relocation, department culture, and personal circumstances. The financial burden of healthy foods was an external factor that likely hindered program compliance. The Recharge360 program promotes a healthy diet by providing a nutrition guide, grocery list and a healthy breakfast, lunch and dinner recipes for each day during the 5-day Recharge week. Respondents highlighted the financial burden of buying healthy food products (i.e., fresh fruit and vegetables), particularly for PhD students and some healthcare workers. It was pointed out that healthy foods are generally more expensive. Additionally, one respondent pointed out that government policies in the Netherlands can be seen as promoting unhealthy eating habits, making it more difficult to eat healthy. As one respondent noted: “They're supposed to eat healthy, but they must pay for that healthy food. That might be a problem in our department, especially for the junior researchers, but it's even more of an issue in the healthcare department. The government promotes unhealthy eating, so that's quite difficult.”

In addition, during the recruitment phase of the pilot study, the participating research department relocated to another Amsterdam UMC location. This hindered the promotion of the program and recruitment of participants due to increased remote work and reduced interactions between colleagues, leading to less word-of-mouth promotion.

In contrast to the facilitating role it played in some departments, department culture was a barrier for some participants. The busy nature of the departments and the rapid pace of e-mail communication contributed to messages being overlooked or quickly deleted, hindering program compliance. Balancing participation in the Recharge360 program with work commitments posed another challenge. Some were hesitant to commit to the program, because of their already demanding work schedules.

Finally, personal circumstances, such as family circumstances or work commitments, sometimes influenced participants' ability to fully engage with the program. Despite these challenges, many participants expressed a willingness to explore ways to enhance their well-being and vitality.

1. Implementation - fidelity

*2.1.* *Delivery of the program according to protocol*

The pilot program was largely implemented in accordance with the protocol. This included recruitment of participants via the administration office of the two participating departments. The content, frequency, duration of the Recharge weeks and the administration of questionnaires were in line with the protocol. We deviated from the protocol by adding additional recruitment strategies, including distributing flyers in the participating departments, short presentations at department activities and an article in the department newsletter. According to the protocol, physical activity would be measured via a step counter in the Recharge360 app for a subgroup of participants who owned an activity tracker (e.g., Fitbit or Apple Watch) by linking their device to the app. Although the step counter was visible in the app, technical issues with this function prevented successful linkage of activity trackers and thus this function was not properly implemented.

*2.2. Facilitators and barriers related to fidelity in the pilot implementation*

Identified facilitators related to fidelity included app functionality and user experience, valuable program content, accessibility of program components, team aspect and competition, clear information materials and communication. Regarding app functionality and user experience, the app was perceived as user-friendly, with a clear and attractive interface that made it easy to fill in the completed tasks. According to most participants, the app generally worked well, despite the issue with the step counter (see the barrier ‘Technical issues application’ below). Also, Respondents highlighted several positive aspects regarding valuable program content. They appreciated the reminders to engage in healthy habits, such as drinking enough water and avoiding snoozing. These simple, actionable tips were beneficial and easy to incorporate into daily routines. Some participants also felt encouraged to reflect on their lifestyle choices and provided opportunities for mindfulness and gratitude, which were particularly valuable during busy periods. This increased awareness helped individuals prioritize self-care. Engaging in healthy habits during the program prompted some individuals to continue these practices beyond their duration. Furthermore, around half of the respondents found the program components (e.g., most tasks and challenges) accessible and not overly demanding, making them manageable for individuals with varying fitness levels and lifestyles.

Regarding the team aspect and competition, the team-based activities fostered a sense of collaboration and motivation for multiple participants. Collaborating with colleagues created a supportive environment where participants encouraged each other to complete tasks and achieve goals. The competitive aspect of the program, such as earning points and participating in a team, added an element of fun and motivation for some participants. They felt that this friendly competition increased engagement among them. One respondent noted: “I really enjoyed that we were all involved together, not exactly a competition, but working on it collectively. I believe we were also the winning team in the department, with the most points. I really liked that, it was a fun element, and I also heard from other people that they found it fun too. It also motivated us to do things because if someone said, ‘Yeah, I did this,’ then I'd think, ‘Shoot, I'm falling behind.”

Lastly, clear information materials and communication were appreciated. Although respondents mentioned that the information materials provided were lengthy, according to other they were also clear and understandable. Some appreciated the elaborate information and found it transparent, helping them feel well-informed about what was expected of them, and it ensured all relevant details were included. The recruitment process was described as low-pressure and easy to engage with. Those who knew about the ability to enable notifications found the reminders effective to keep them on track.

Identified barriers related to fidelity included repetitiveness of program components, practical issues and time investment for cooking recipes and mindfulness exercises, unrealistic program components and competition, anonymity, amount and clearness of information, and technical issues with the application. Some participants reported a decline in motivation due to repetitiveness of program components, such as repeated daily activities and a lack of automatic variation in the sport or yoga videos and mindfulness exercises. They expressed a desire for more diverse content and felt the program would be more effective if it aligned better with their specific needs and schedules, such as allowing flexibility in choosing activities and the weeks in which they wanted to follow the program. One respondent noted: “The first time it was fun, and you are curious, and then the second and third times you think, it was the same each time. I think that's why you engage fewer people to keep it up.”

Additionally, practical issues and time investment for cooking recipes and mindfulness exercises posed challenges. Respondents particularly found the recipes for breakfast and lunch difficult to integrate into their routines. Recommendations included varied simpler family-friendly recipes. One respondent noted: “I noticed that because I thought I was going for it, during that first week I was spending at least an hour in the kitchen every evening to prepare lunch or things for dinner and such. And then you realize, even though you want to do it, if it takes so much time, at some point I started to feel resistance, especially in combination with all the other things you already do.” Another respondent noted: “I did find that healthy eating was sometimes difficult because you’d get a recipe and you don't always have the ingredients at home, or you can’t always go to the supermarket. For example, I often do groceries once or twice a week, enough for the whole week, and if you haven't received the recipe yet, it's hard to prepare it.” Also, for some participants mindfulness exercises, while initially engaging, lost their appeal due to time constraints and impracticality in a work environment without private space.

Regarding unrealistic program components and competition, tasks like early morning physical activity and cold showers were seen as unrealistic by a few participants, which was experienced as demotivating. Recommendations included a more personally tailored program, fostering small steps in behavioral change, considering the starting point of each participant. One respondent noted: “If people hardly exercise and you force them to do those fifteen minutes every morning or things like that, you know… I’m often bad at drinking enough, especially at home. So to say, ‘I drink this many glasses’, no, that shouldn’t be the challenge. The challenge should be something like: ‘This week, challenge yourself to drink at least two glasses before noon every day.’ And then you build on that. If someone says, ‘Yeah, I can manage that, it’s becoming part of my routine,’ then you add a new challenge. So, in addition to those two glasses before noon, now also drink two more before 3 PM. That way, you gradually create behavioral change that sticks.”

Although the team aspect of the program was voluntary and the point system and competitive elements between teams were motivating for most, one participant felt pressured to achieve high scores and experienced feelings of guilt, leading to demotivation. Recommendations included emphasis on personal progress and encouragement over competition. As one respondent said: “Yes, but that's also because you try to have a bit of a competition, with a score. That also contributes to the feeling of guilt because I'm skipping a lot.”

Anonymity also arose as a barrier, as all e-mails, communication and names in the team competition were anonymized, colleagues often did not know which other colleagues participated. Also, within the team page, participants could not see each other names once in the team or that of other participants from other teams. The anonymity of the program and therefore lack of communication among participants hindered collaboration and engagement in the program.

Furthermore, the amount and clearness of information posed challenges. Some participants found it challenging to discover all the available resources, leading to the underutilization of these features. Also, it was not clear to some participants that the program design allowed participants to choose parts that worked for them and skip others, causing some respondents to feel guilty for skipping tasks. Finally, the recruitment and information materials were often considered too lengthy, and respondents preferred concise, step-by-step instructions with visuals. Some participants were unaware of how to enable notifications and reminders, which affected their engagement with the program. One respondent said: “You always have to be careful about an overload of information. I found the information we received about the challenge, at least my first impression and my impressions are always very quick because I don’t have much time, quite a lot of information, which made me think, "What is this?" Ideally, I want to see immediately what it's about, kind of in a marketing way, which might make you more inclined to say yes even faster.”

Finally, some technical issues with the application arose. The Recharge360 app included a step counter that could be linked to the activity tracker of participants. Although the step counter was already visible in the app, it did not work properly yet, because participants could not link their activity trackers to the app and could thus not use the step counter. This demotivated some participants. Most participants did not report additional problems beyond the malfunctioning step counter. Some participants encountered difficulties with the app installation and activation, contributing to a less smooth experience.

1. Implementation - dose

*3.1.* *Quantity of the program delivered and received*

The same quantity of the program was delivered to all participants, including a motivational information session before the start of the program, and three Recharge weeks of 5 days including all program components. However, the dose received, referring to the amount of Recharge weeks actually participated in as well as the amount of program components followed/ degree of participation differed between participants, as all Recharge weeks and program components were voluntary.

While the dose delivered remained consistent throughout the pilot study, the actual dose received by participants declined over time. Regarding participation in the Recharge360 program, of the 35 participants that signed up to participate, 29 (83%) participants started the first Recharge week, 18 (51%) participants started the second Recharge week, and 12 (34%) participants started the third Recharge week. 11 (31%) participants followed the complete program, including all three Recharge weeks (Table 5). For the participants that started a Recharge week, the degree of participation (reflected by the mean score per week) declined mainly in the office program, as illustrated by lower median scores across the Recharge Weeks, with the lowest scores in the third week. However, participation in the healthcare program remained relatively stable, even peaking in the second week.

Table 5. Degree of participation in the Recharge weeks.^a^

|  | **Recharge week 1** | | **Recharge week 2** | | **Recharge week 3** | |
| --- | --- | --- | --- | --- | --- | --- |
| **Program** | **Median score** | **n** | **Median score** | **n** | **Median score** | **n** |
| **Both** | 51 [20, 65] | 29 | 49 [20, 66] | 18 | 40 [26,49] | 12 |
| **Healthcare** | 42 [19, 57] | 8 | 54 [32, 65] | 3 | 41 [32, 44] | 3 |
| **Office** | 55 [ 20, 77] | 21 | 46 [21, 64] | 15 | 38 [27, 58] | 9 |
| 1. In people that started at least one Recharge week. 2. The maximum score that could be obtained for one Recharge week was week was 196 (healthcare) or 200 (office). | | | | | | |

*3.2. Facilitators and barriers related to dose in the pilot implementation*

Identified facilitators related to dose included a suitable duration of activities and questionnaires,

Good frequency of communication materials, and well-distributed activities. Some participants highlighted the suitable duration of activities and questionnaires, noting that the time required to complete the questionnaires was manageable. The questionnaires were convenient to fill out on the go or during short breaks. Similarly, the time commitment for the activities was considered appropriate by some. Furthermore, the frequency of e-communication (e.g., mails and announcements) was deemed satisfactory by some. The two-week interval between the first e-mails and the registration for participation provided enough time for participants to read through the information and consider their involvement in the program. Finally, one respondent particularly appreciated the distribution of activities into morning, afternoon and evening routines. This division allowed them to update and fill in information during short breaks throughout the day, making it easier to integrate the tasks into their daily routine. The respondent noted: “I liked that it was divided into three parts: morning, afternoon, and evening. That allows you to update and input things in between. I prefer that rather than having to fill in everything at the end of the day. So, I also received occasional notifications like, ‘Now it's afternoon, time for healthy eating or exercising, etc.”

Identified barriers related to dose included the spread of Recharge weeks, a lack of (personal) communication in between Recharge weeks and lengthy activities and questionnaires. Regarding the spread of the Recharge weeks, the long gap between the Recharge weeks made it challenging to stay engaged for some participants. Some participants indicated that they would have preferred the program weeks to be closer together. Additionally, during the breaks between the Recharge weeks, some participants expressed a desire for additional communication. One respondent felt unsure about whether to continue certain practices or potentially missing any instructions. Furthermore, one respondent felt that the e-mails were too automated and lacked a personal touch. Lastly, one participant mentioned the kick-off meeting was too long (45 minutes), suggesting it could have been shorter and more concise. Some considered the questionnaires too lengthy. Over time, it became harder for participants to invest as much time in the activities as they did initially, and some found the tasks to be too time-consuming or difficult to fit into their schedules.

1. Implementation - reach

*4.1.* *Audience reached and characteristics of the recruited participants*

Participants that were recruited in the pilot study (n=34), including 9 employees of a healthcare department and 25 of a research department, were mostly female (88%), were mostly highly educated (85%), had a slightly higher t-score for global physical health (50.5) and mental health (47.1) compared to the general Dutch population (45.2 and 44.7, respectively)[4], mostly never smoked (88%) and on average had a normal BMI (23.4 ±3.0 kg/m^2^)(Table 6). Regarding the research department, people with diverse professions were reached, while for the healthcare department there is less variation (e.g. only one nurse was reached and no laboratory staff) (Table 4). Together, this indicates that quite a selective group was reached.

Table 6. Baseline characteristics pilot group.

|  | Pilot population (n=34) |
| --- | --- |
| Demographics  Age, years  Women, %  High education level, % | 37 ± 11.2  30 (88.2)  29 (85.3) |
| Other characteristics  Children at home (yes), %  Working hours, %  <24 hours  25-36 hours  >36 hours  High stress, %  Global physical health, t-score  Global mental health, t-score  Self-rated health, %  Poor  Fair or good  Very good or excellent | 14 (41.2)  4 (11.8)  14 (41.2)  16 (47.1)  50.5 ±4.56  47.1 ±3.66  0 (0)  14 (41.2)  20 (58.8) |
| Well-being  WHO-5, % score (range 1-100) | 60 ±14 |
| Work-related vitality  Work ability, score (range 1-10)  Sick days last 5 months (≥9 days), %  Need for recovery  (range 1-100), score  Task performance  (range 1-4), score | 8 ±1.2  1 (2.9)  37.2 ±28.5  2.3 ±1.0 |
| Lifestyle  Smoking behavior, %  Current  Former  Never  Electronic smoking behavior, %  Never  BMI, kg/m^2^  Vegetable intake (≥200g/day), %  Fruit intake (≥2 pieces/day), %  Alcohol intake (>7 glasses/week),%  Sleep duration (7-9 hours/day), %  Overall sleep quality (Fairly good or very good), %  Physical activity,  MET minutes/week^a^ | 1 (2.9)  3 (8.8)  30 (88.2)  34 (100)  23.4 ±3.0  5 (14.7)  9 (26.5)  5 (14.7)  26 (76.5)  29 (85.3)  2358 [1524, 4013]^a^ |
| Categorical variables are presented as frequencies (%) and continuous variables as mean ± standard deviation if normally distributed or as median [interquartile ranges (IQR)] if non-normally distributed after visual assessment of histograms.  a. The analytic sample was n=27 for MET minutes/week.  Abbreviations: WHO, World Health Organisation; BMI, Body Mass Index; MET, Metabolic Equivalent of Task. | |

*4.2. Facilitators and barriers related to reach in the pilot implementation*

A key facilitator related to reach was contact with the project team and departmental e-mails. Participants most often heard about the Recharge360 program and pilot through contact with the project team (especially for the research department) and departmental e-mails, which were appreciated. Although most participants were recruited via contact with the project team and departmental e-mails, flyers included in the Christmas packages and mentions during departmental seminars were perceived as effective strategies for promoting the program by some participants.

On the other hand, a key barrier related to reach were passive and formal recruitment strategies. There were also a few participants that perceived the recruitment strategies as too passive or formal, which might be insufficient for generating enthusiasm for a larger audience. One participant noted that, although the program was mentioned in staff meetings, active promotion by departments heads could have increased participation and that utilizing informal and formal settings (e.g., various section meetings), would make announcements more engaging and less formal. Also, emphasizing the personal and team benefits of participation could create a stronger motivation for engagement. For example, “Encouraging participation could perhaps have been done more actively. We have a seminar once every two weeks, and it would be beneficial if, after a presentation, you reminded everyone again after two weeks or a month by saying, ‘Hey guys, don't forget about this.”

1. Implementation - adaptations

A few adjustments were made during the pilot study to better fit different contexts or to address participants’ needs following feedback during the pilot. During the recruitment phase of the pilot study, the participating research department relocated to another Amsterdam UMC location. Therefore, it was not possible to promote the study by physical posters at the research department, leading to an adapted recruitment strategy for this department, including a pitch by the project team at the department seminar, an article in the department newsletter and a short pitch at the department Christmas activity. To address repetitiveness of program components, the daily exercises in the dashboard were varied over the Recharge period, offering different exercises within the same category from the Recharge360 app repository. To address a lack of communication between Recharge weeks, an extra e-mail was sent in between Recharge weeks to engage participants. Finally, one participant from the healthcare department switched from the healthcare program to the office program because this participant had an office job within the healthcare department.

1. Other factors influencing implementation

*6.1.* *Personal reasons to participate*

The participants' motivations for participating in the Recharge360 program and pilot study varied, but common themes emerged. Many saw it as an opportunity for improving lifestyle behaviors. Some felt a sense of obligation to participate due to their roles within the department or to show support for the project team or to support colleagues in research efforts. Others joined out of curiosity or to learn more about topics related to health and well-being. For example, one participant mentioned: “Well, I already move quite a lot on my own, but I thought maybe it would be nice to gain a new perspective and because my boss initiated it. I always think it's good to participate, more support from the group. Then I feel like I should participate myself, if I maintain all the contacts, send e-mails. I didn't feel forced, but slightly compelled.” Another respondent noted: “I was really looking forward to it. I thought it all sounded good. Vital employees and of course; everyone wants to be vital at work. I thought I can probably learn something from it too, like a bit of mindfulness. I had a bit of a picture in my mind of, okay, then you integrate some positive habits into your routine, and that app would help me integrate those positive habits. So that's why I joined; I did feel a need for that.”

*6.2. Other facilitators: easy and valuable contact with the project team*

The participants generally found the communication with the project team to be straightforward and accessible. They appreciated the ease of contacting the team via e-mail and receiving prompt responses. Overall, the interactions with the team were seen as positive and helpful in facilitating their participation in the program.

1. Improvements to the main study

The results of the pilot process evaluation were used to improve the implementation of the subsequent larger-scale main study. The most important changes included modifications to the recruitment process, which no longer targeted individual departments but instead encompassed the entire Amsterdam UMC through the use of internal digital news articles, flyers at diverse locations, posters, and the possibility to invite colleagues. Recruitment via department administration offices was not feasible for scaling up to the entire Amsterdam UMC. Also, the tone and design of the informational materials were clarified, with more pictures and a step-by-step guide.

Regarding the Recharge360 program itself, there was a reduced emphasis on making three recipes a day, as this was not feasible for most participants; instead a recipe for lunch was provided daily on the dashboard, and recipes for breakfast and dinner were optional. More variety in exercises was provided, and exercises were formulated shorter and distributed more evenly throughout the day to prevent overload and a better balance of daily activities. Furthermore, the team element of the program was promoted more effectively, with increased emphasis on performing activities with colleagues. Based on participant needs, additional functionalities were introduced, such as the option to record personal physical activities within the app. Lastly technical issues were resolved, such as the dysfunctional step counter.

**References**

1. Craig P, Dieppe P, Macintyre S, Michie S, Nazareth I, Petticrew M. Developing and evaluating complex interventions: the new Medical Research Council guidance. BMJ. 2008;337:a1655. PMID:18824488 doi: 10.1136/bmj.a1655

2. Moore GF, Audrey S, Barker M, Bond L, Bonell C, Hardeman W, et al. Process evaluation of complex interventions: Medical Research Council guidance. BMJ: British Medical Journal. 2015;350:h1258. PMID:25791983 doi: 10.1136/bmj.h1258

3. VERBI Software. MAXQDA 2022. Berlin, Germany. 2021.

4. Elsman EB, Roorda LD, Crins MH, Boers M, Terwee CB. Dutch reference values for the Patient-Reported Outcomes Measurement Information System Scale v1. 2-Global Health (PROMIS-GH). Journal of Patient-Reported Outcomes. 2021;5(1):1–9. PMID:33978855 doi: 10.1186/s41687-021-00314-0
