# Supplementary material for: The Feasibility of an App-Based Worksite Health Promotion Program to Improve Mental Well-Being and Work-Related Vitality in University Hospital Workers: Process and Preliminary Effect Evaluation Study
Source: JMIR Form Res. 2026 Jun 17;10:e85135. doi: 10.2196/85135 (PMC13274912; doi:10.2196/85135)
Supplement: Multimedia Appendix 2 [file formative-v10-e85135-s002.docx]

**Appendix 3**

**Supplemental methods Recharge360 application**

The Recharge360 app consists of five pages (Supplemental figure 1), of which three main pages, namely the dashboard, media and team.


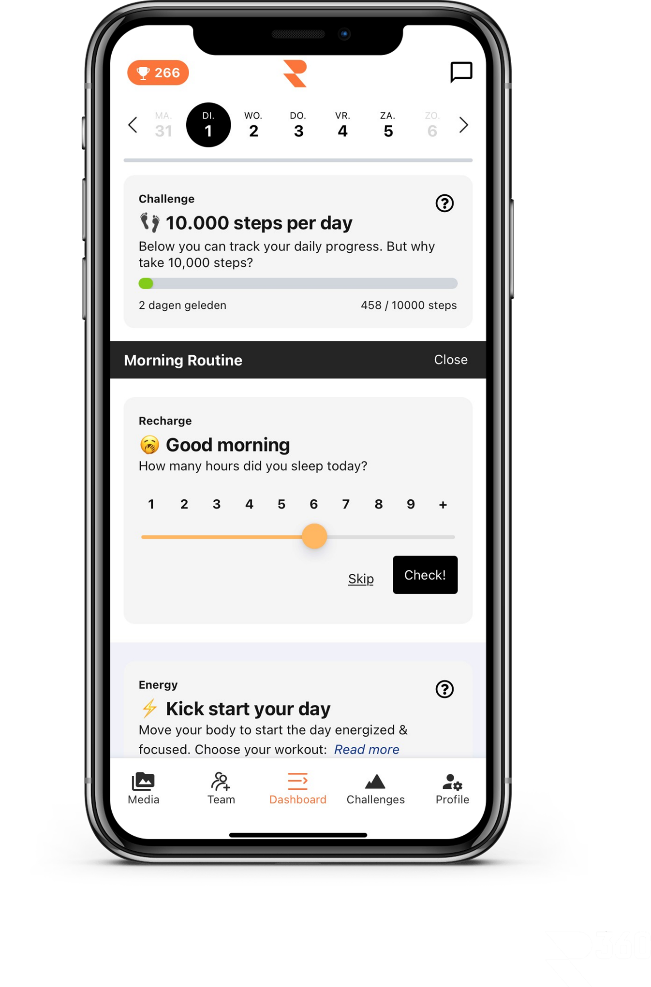

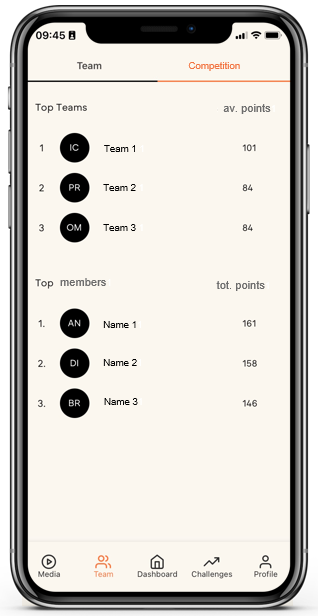

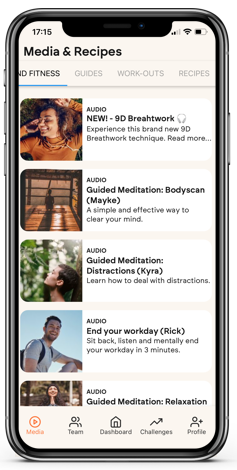


Supplemental figure 1. Three main pages of the Recharge360 application: dashboard, media and team.

*Dashboard*

For each day separately (Monday to Friday), the program contained returning advice every day, such setting priorities and a short physical exercise in the morning, preparing a healthy lunch (recipes provided) in the afternoon, and going to bed in the evening in time to get 8 hours of sleep. Next to this, the program contained varying exercises and challenges focusing on multiple lifestyle components, including nutrition, alcohol use, physical activity, relaxation (mental balance), stress, and sleep and tailored to healthcare or office workers. Participants could earn points by completing program components. Some examples are, eating two pieces of fruit, drinking no alcohol, preparing a healthy lunch (recipes provided), finishing a physical, mindfulness or focus exercises or using no screens before bedtime. During five days a total of 164 points could be earned 164 for the healthcare program and 161 for the office program.

*Media*

The media library contained: 1) mindfulness exercises (videos), 2) physical exercises (videos), 3) recipes for a healthy breakfast, lunch and dinner, and 4) supportive guides, such as an evidence-based nutrition guide based on the Dutch dietary guidelines (ref), a grocery list, additional advice for night workers and advice on how to follow the program as a parent.

*Team*

Participants could voluntarily participate in a team competition with colleagues. The page displayed the total score of each Amsterdam UMC team participating in the Recharge360 program as well as the ranking of participants within a team. The total team score was calculated based on the individual scores of each team participant. In this way, each individual´s use of the program benefited the whole team. At the end of the program, the team scoring most points were announced.
