# Supplementary material for: The Feasibility of an App-Based Worksite Health Promotion Program to Improve Mental Well-Being and Work-Related Vitality in University Hospital Workers: Process and Preliminary Effect Evaluation Study
Source: JMIR Form Res. 2026 Jun 17;10:e85135. doi: 10.2196/85135 (PMC13274912; doi:10.2196/85135)
Supplement: Multimedia Appendix 3 [file formative-v10-e85135-s003.docx]

**Appendix 4**

**Supplemental methods measurement of sociodemographic, lifestyle and other characteristics**

At baseline, sociodemographic and other characteristics were assessed with a self-report questionnaire, including age (years), sex (man, woman, other), education level (low, middle, high), household (1, 2, 3, >3 persons), children at home (yes, no), pregnant (yes, no), working hours (8-16, 17-24, 25-36, >36 hours), sickness absenteeism over the past 5 months (0, <9 or ≥10 sick days), experienced stress (high, low), global health (t-scores), self-rated health (poor, fair, good, very good, excellent) and shift work (rotating shifts, night shifts, neither). As previously described by Kouwenhoven-Pasmooij et al. [1] in the PerfectFit study, perceived stress was measured by using INTERHEART questionnaire [2]. We defined “high stress level” as some-several periods or permanent stress at work or home, severe financial stress and/or 2 or more stressful life events in the past year [1, 2]. Global health was assessed with the Patient-Reported Outcome Measurement Information System Scale v1.2 - Global Health (PROMIS-GH)[3, 4]. The PROMIS-GH consists of ten items, of which eight items are divided into two subscales: 1) global mental health, 2) global physical health. Total scores were calculated based on the original US item response theory (IRT) model and expressed as T-scores with a mean ± standard deviation of 50 ± 10 in the general US population. Higher scores indicate better global mental or physical health. The PROMIS-GH subscales showed acceptable structural validity, internal consistency and cross-cultural validity in the Dutch general population [3, 4]. The t-score in the Dutch general population is 44.7 ± 8.0 for global mental health and 45.2 ± 9.2 for global physical health [4]. Self-rated health was assessed with a separate item of the PROMIS-GH.

Lifestyle factors were assessed with a self-report questionnaire, including weight (in kg), fruit intake (<2 or ≥2 pieces per day), vegetable intake (<200 or ≥200 grams per day), alcohol intake, smoking habits (current, former, never), sleep duration between seven to nine hours (yes/no), sleep quality (very good, fairly good, rather poor, very poor ) and physical activity (≤7 or >7 glasses per week).

Self-reported body weight in kilograms was assessed as weight after getting up in the morning, in a fasted state, with light clothing. A short nutrition screener assessed compliance with the Dutch Healthy Dietary Guidelines 2015 regarding fruit intake, vegetable intake and alcohol consumption. The screener assessed daily self-reported intake of vegetables on a 6-point scale (“no vegetables” to “4 or more servings spoons of 50 grams per day”), and fruit on a 7-point scale (“(almost) never” to “three or more times a day) over the past 2 weeks. The recommendation was not met if less than 200 grams of vegetables and less than 2 pieces of fruit were consumed daily. Alcohol intake was assessed by first asking how many days per week alcohol is consumed ('I never drink alcoholic beverages' to '7 days') and then how many glasses are consumed on average in a day ('1' to '7 or more'). For more than seven glasses per week, the guideline was not met. Smoking habits were assessed as never, former or current tobacco use and use of (electronic) cigarettes. Sleep quality was measured with two items from the Pittsburgh Sleep Quality Index (PSQI)*[5]*. The first item measures sleep duration and the second item measures overall sleep quality over one month (very good, fairly good, rather poor, very poor) [6, 7]. Sleep duration was divided into two categories (meet sleep recommendations vs. less than or more than sleep recommendations), based on recommendations for optimal amount of sleep (7 to 9 hours per) according to National Sleep Foundation guidelines [8]. Sleep quality was also divided into two categories (very good vs. fairly good, rather poor, very poor). Although the PSQI is a validated and reliable measure for sleep quality in various samples, these items are not validated to be used separately [5]. Physical activity was assessed with the International Physical Activity Questionnaire short form (IPAQ-SF), quantifying physical activity over the last seven days into four categories: high-intensity physical activity (VPA), moderate-intensity physical activity (MPA), walking and sitting [9]. Metabolic equivalent of task (MET) in minutes/week is calculated according to established methods [9]. The validity of the IPAQ-SF is considered weak as compared to objective measures of physical activity, for various populations, although the reliability is shown to be acceptable to be used with in repeated measures studies [9].

**References**

1. Kouwenhoven-Pasmooij TA, Robroek SJW, Kraaijenhagen RA, Helmhout PH, Nieboer D, Burdorf A, et al. Effectiveness of the blended-care lifestyle intervention 'PerfectFit': a cluster randomised trial in employees at risk for cardiovascular diseases. BMC Public Health. 2018;18(1):766. PMID: 29921255. doi: 10.1186/s12889-018-5633-0.

2. Rosengren A, Hawken S, Ounpuu S, Sliwa K, Zubaid M, Almahmeed WA, et al. Association of psychosocial risk factors with risk of acute myocardial infarction in 11119 cases and 13648 controls from 52 countries (the INTERHEART study): case-control study. Lancet. 2004;364(9438):953–62. PMID: 15364186. doi: 10.1016/s0140-6736(04)17019-0.

3. Elsman EB, Roorda LD, Crins MH, Boers M, Terwee CB. Dutch reference values for the Patient-Reported Outcomes Measurement Information System Scale v1. 2-Global Health (PROMIS-GH). Journal of Patient-Reported Outcomes. 2021;5(1):1–9. PMID: 33978855. doi: 10.1186/s41687-021-00314-0.

4. Pellicciari L, Chiarotto A, Giusti E, Crins MH, Roorda LD, Terwee CB. Psychometric properties of the patient-reported outcomes measurement information system scale v1. 2: global health (PROMIS-GH) in a Dutch general population. Health and quality of life outcomes. 2021;19(1):1–17. PMID: 34579721. doi: 10.1186/s12955-021-01855-0.

5. Buysse DJ, Reynolds CF, Monk TH, Berman SR, Kupfer DJ. The Pittsburgh sleep quality index: A new instrument for psychiatric practice and research. Psychiatry Research. 1989 1989/05/01/;28(2):193–213. PMID: 2748771. doi: 10.1016/0165-1781(89)90047-4.

6. Hinz A, Glaesmer H, Brähler E, Löffler M, Engel C, Enzenbach C, et al. Sleep quality in the general population: psychometric properties of the Pittsburgh Sleep Quality Index, derived from a German community sample of 9284 people. Sleep Medicine. 2017;30:57–63. PMID: 28215264. doi: 10.1016/j.sleep.2016.03.008.

7. Smyth C. The Pittsburgh sleep quality index (PSQI). SLACK Incorporated Thorofare NJ; 1999. p. 10.

8. Hirshkowitz M, Whiton K, Albert SM, Alessi C, Bruni O, DonCarlos L, et al. National Sleep Foundation’s updated sleep duration recommendations: final report. Sleep Health. 2015;1(4):233–43. PMID: 29073398. doi: 10.1016/j.sleh.2015.10.004.

9. Lee PH, Macfarlane DJ, Lam TH, Stewart SM. Validity of the international physical activity questionnaire short form (IPAQ-SF): A systematic review. International Journal of Behavioral Nutrition and Physical Activity. 2011;8(1):115. PMID: 22018588. doi: 10.1186/1479-5868-8-115.
