# Supplementary material for: The Feasibility of an App-Based Worksite Health Promotion Program to Improve Mental Well-Being and Work-Related Vitality in University Hospital Workers: Process and Preliminary Effect Evaluation Study
Source: JMIR Form Res. 2026 Jun 17;10:e85135. doi: 10.2196/85135 (PMC13274912; doi:10.2196/85135)
Supplement: Multimedia Appendix 4 [file formative-v10-e85135-s004.docx]

**Appendix 2**

Supplemental table 1. Reasons for lost to follow-up (n=29).

| **Reason** | **N** |
| --- | --- |
| Issues with timing of the program  (e.g., Planned a holiday, participation in another program, too little time to participate) | 7 |
| Personal circumstances  (e.g., informal care) | 6 |
| Technical problems with the Recharge360 app | 4 |
| Did not like program style or content  (e.g., communication style of the program, too little variation in program components, does not fit daily rhythm) | 4 |
| (Long-term) sickness | 4 |
| Program did not meet expectations | 2 |
| Decreased active participation of colleagues | 1 |
| Just don’t feel like it anymore | 1 |
| Program does not lead to the desired effect and notifications on phone are distracting | 1 |
| Not feasible with young children | 1 |
