# Supplementary material for: The Feasibility of an App-Based Worksite Health Promotion Program to Improve Mental Well-Being and Work-Related Vitality in University Hospital Workers: Process and Preliminary Effect Evaluation Study
Source: JMIR Form Res. 2026 Jun 17;10:e85135. doi: 10.2196/85135 (PMC13274912; doi:10.2196/85135)
Supplement: Multimedia Appendix 5 [file formative-v10-e85135-s005.docx]

**Appendix 5**

Supplemental table 2. Comparison of participants who completed the whole program vs. participants who dropped out.

|  | Drop outs - no Recharge weeks  (n=43) | Drop outs - at least 1 Recharge week  (n=345) | Complete program  (n=144) |
| --- | --- | --- | --- |
| Demographics  Age, years  Women, %  High education level, % | 43 ±12  38 (88.4)  35 (81.4) | 43 ±12  314 (91.0)  311 (90.1) | 43 ±12  130 (90.3)  134 (93.1) |
| Other characteristics  Children at home (yes), %  Working hours, %  ≤24 hours  25-36 hours  >36 hours  Type of work, %  Office workers  Healthcare workers  High stress, %  Global physical health, t-score  Global mental health, t-score  Self-rated health, %  Poor  Fair or good  Very good or excellent | 25 (58.1)  7 (16.3)  24 (55.8)  12 (27.9)  NA  NA  30 (69.8)  48.9 ±4.53  46.3 ±3.70  0 (0.0)  29 (67.4)  14 (32.6) | 152 (44.1)    36 (10.4)  240 (69.6)  69 (20.0)  241 (69.9)  104 (30.1)  172 (49.9)  49.7 ±4.55  46.8 ±3.70  1 (0.3)  215 (62.3)  129 (37.4) | 70 (48.6)  13 (9.0)  98 (68.1)  33 (22.9)  119 (82.6)  25 (17.4)  60 (41.7)  50.9 ±4.59  47.3 ±3.69    0 (0.0)  84 (58.4)  60 (41.6) |
| Well-being  WHO-5, % score (range 1-100) | 56 ±18 | 60 ±16 | 60 ±15 |
| Work-related vitality  Work ability, score (range 1-10)  Sick days last 5 months (≥9 days), %  Need for recovery  (range 1-100), score  Task performance  (range 1-4), score | 8 ±2.1  2 (4.7)  53.1 ±32.4  2.5 ±0.7 | 8 ±1.3  19 (5.5)  41.8 ±29.1  2.5 ±0.8 | 8 ±1.2  3 (2.1)  38.4 ±29.3  2.5 ±0.7 |
| Lifestyle  Smoking behavior, %  Current  Former  Never  Electronic smoking behavior, %  Current  Former  Never  BMI, kg/m^2 a^  Vegetable intake (≥200g/day), %  Fruit intake (≥2 pieces/day), %  Alcohol intake (>7 glasses/week),%  Sleep duration (7-9 hours/day), %  Overall sleep quality (fairly good or very good), %  Shifts during the past month, %^a^  Rotating and/ or night shifts  Neither  Physical activity,  MET minutes/week^a^ | 3 (7.0)  6 (14.0)  34 (79.1)  0 (0.0)  2 (4.7)  41 (95.3)  25.1 ±4.9  12 (27.9)  17 (39.5)  10 (23.3)  28 (65.1)  26 (60.5)  9 (20.9)  34 (79.1)  2360 [1082, 3107] | 19 (5.5)  78 (22.6)  248 (71.9)  7 (2.0)  12 (3.5)  326 (94.5)  25.0 ±4.8  100 (29.0)  123 (35.7)  42 (12.2)  248 (71.9)  269 (77.9)  41 (11.9)  304 (88.1)  2109 [1189, 3555] | 4 (2.8)  36 (25.0)  104 (72.2)  0 (0.0)  3 (2.1)  141 (97.9)  24.4 ±4.5  41 (28.5)  50 (34.7)  16 (11.1)  105 (72.9)  108 (75)  11 (7.7)  132 (92.3)  2132 [1110, 3714] |
| Categorical variables are presented as frequencies (%) and continuous variables as mean ± standard deviation if normally distributed or as median [interquartile ranges (IQR)] if non-normally distributed after visual assessment of histograms.  Abbreviations: WHO, World Health Organization; BMI, Body Mass Index; MET, Metabolic Equivalent of Task.  a. The analytic sample was n=528 for BMI, n=531 for shifts during the past month and n=369 for MET minutes/week. | | | |
